# Supplementary material for: Circulating Myeloperoxidase (MPO)-DNA complexes as marker for Neutrophil Extracellular Traps (NETs) levels and the association with cardiovascular risk factors in the general population
Source: PLoS One. 2021 Aug 11;16(8):e0253698. doi: 10.1371/journal.pone.0253698 (PMC8357174; doi:10.1371/journal.pone.0253698)
Supplement: S1 File — S1 Table. Correlations between MPO-DNA complex and clinical characteristics. S2 Table. MPO-DNA complex levels in CVD risk factors. S3 Table. Correlations between MPO-DNA complex and inflammatory and immunology markers in a subset of 1208 individuals of RS-I-3. S1 Fig. Circadian rhythm of MPO-DNA complex levels during daytime. S2 Fig. Typical example of a reference curve used for MPO-DNA complex ELISA. S3 Fig. Correlation between MPO-DNA complex and citrullinated histone H3. (DOCX) [file pone.0253698.s001.docx]

***Circulating Myeloperoxidase (MPO)-DNA complexes as marker for neutrophil extracellular traps (NETs) levels and the association with cardiovascular risk factors in the general population***

Samantha J. Donkel, MD^1^, Frank J. Wolters, MD, PhD^2,3^, M. Arfan Ikram, MD, PhD^2^, Moniek P.M de Maat, PhD^1^.

^1^Department of Hematology, Erasmus University Medical Center, Rotterdam, the Netherlands.
^2^Department of Epidemiology, Erasmus University Medical Center, Rotterdam, the Netherlands.
^3^Department of Radiology & Nuclear Medicine, Erasmus University Medical Center, Rotterdam, the Netherlands.

**S1 Table. Correlations between MPO-DNA complex and clinical characteristics.**

|  | Correlation coefficient | p-value |
| --- | --- | --- |
| Age (years) | -0.07 | <0.01 |
| Sex (male versus female) | -0.02 | 0.12 |
| Current smoking (current versus never) | -0.00 | 0.80 |
| BMI (kg/m^2^) | 0.02 | 0.12 |
| Systolic blood pressure (mmHg) | -0.03 | 0.03 |
| Hypertension | -0.04 | <0.01 |
| Diabetes Mellitus | -0.02 | 0.08 |
| History of CVD  - History of CHD  - History of stroke | -0.03  -0.04  -0.00 | 0.01  <0.01  0.92 |
| Antithrombotic medication | -0.02 | 0.08 |
| Lipid-reducing agents | -0.04 | <0.01 |
| *Blood measurements* |  |  |
| Total cholesterol (mmol/L) | -0.00 | 0.76 |
| HDL (mmol/L) | -0.05 | <0.01 |
| Glucose (mmol/L) | -0.01 | 0.63 |
| CRP (mg/L) | -0.01 | 0.66 |
| Leukocytes (*10^-9^/L) | 0.03 | 0.02 |
| Fibrinogen (g/L) | -0.01 | 0.61 |
| VWF (IU/mL) | 0.00 | 0.95 |
| ADAMTS13 (%) | 0.00 | 0.78 |

CVD: cardiovascular disease, CHD: coronary heart disease. HDL: high density lipoprotein, CRP: C-reactive protein, VWF: Von Willebrand Factor, ADAMTS13: a disintegrin and metalloproteinase with a thrombospondin type 1 motif, member 13

**S2 Table. MPO-DNA complex levels in CVD risk factors.**

|  |  | MPO-DNA complex (mAU/mL) | p-value |
| --- | --- | --- | --- |
| Sex | Male (n=2796)  Female (n=3633) | 54 (42-88)  53 (42-85) | 0.13 |
| Current smoking | Yes (n=1111)  No (n=5249) | 53 (43-85)  53 (42-87) | 0.81 |
| BMI, kg/m^2^ | <30 (n=5124)  ≥30 (n=1234) | 53 (42-87)  53 (43-87) | 0.61 |
| Hypertension | Yes (n=4376)  No (n=2019) | 53 (41-85)  55 (44-89) | <0.01 |
| Diabetes Mellitus | Yes (n=751)  No (n=5680) | 52 (41-81)  54 (42-87) | 0.09 |
| History of CHD | Yes (n=413)  No (n=5961) | 51 (38-78)  53 (43-87) | <0.01 |
| History of stroke | Yes (n=254)  No (n=6174) | 53 (43-79)  53 (42-87) | 0.93 |
| Lipid-reducing agents | Yes (n=816)  No (n=5613) | 52 (40-79)  54 (43-88) | <0.01 |
| Total cholesterol, mmol/L | <6.5 (n=4990)  ≥6.5 (n=1437) | 53 (42-87)  53 (42-86) | 0.62 |
| HDL, mmol/L | <1.5 (n=4290)  ≥1.5 (n=2068) | 55 (43-90)  51 (41-80) | <0.01 |

Data are presented as median and 25^th^-75^th^ percentiles. BMI: body mass index, CHD: coronary heart disease, HDL: high-density lipoprotein.

**S3 Table. Correlations between MPO-DNA complex and inflammatory and immunology markers in a subset of 1208 individuals of RS-I-3.**

|  | **Correlation coefficient** | **p-value** |
| --- | --- | --- |
| Complement factor C3 (g/L) | -0.01 | 0.79 |
| IgA (g/L) | -0.07 | 0.02 |
| IgE (g/L) | -0.01 | 0.65 |
| IgM (g/L) | 0.20 | <0.01 |
| IL-1beta (pg/mL) | 0.02 | 0.61 |
| IL-1ra (pg/mL) | 0.02 | 0.58 |
| IL-3 (pg/mL) | -0.05 | 0.12 |
| IL-4 (pg/mL) | -0.08 | <0.01 |
| IL-5 (pg/mL) | -0.06 | 0.03 |
| IL-7 (pg/mL) | -0.01 | 0.64 |
| IL-8 (pg/mL) | -0.01 | 0.75 |
| IL-10 (pg/mL) | -0.01 | 0.87 |
| IL-12p70 (pg/mL) | 0.03 | 0.36 |
| IL-13 (pg/mL) | -0.07 | 0.02 |
| IL-15 (pg/mL) | 0.00 | 0.92 |
| IL-16 (pg/mL) | 0.03 | 0.27 |
| IL-17 (pg/mL) | 0.07 | 0.02 |
| IL-18 (pg/mL) | 0.01 | 0.63 |
| TNFα (pg/mL) | 0.06 | 0.05 |

IL: interleukin, Ig: immunoglobulin, TNF: tumor necrosis factor**.**

**S1 Fig. Circadian rhythm of MPO-DNA complex levels during daytime.**


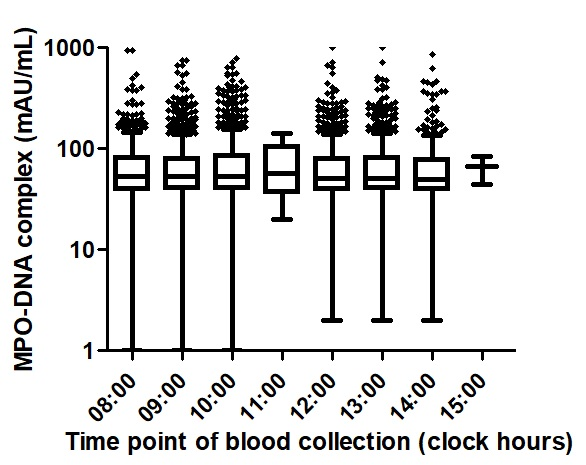


Levels of MPO-DNA complex between 8 AM and 4 PM. Data are presented as median and 25^th^-75^th^ percentiles. The Y-axis was log-transformed.

**S2 Fig. Typical example of a reference curve used for MPO-DNA complex ELISA.**

**
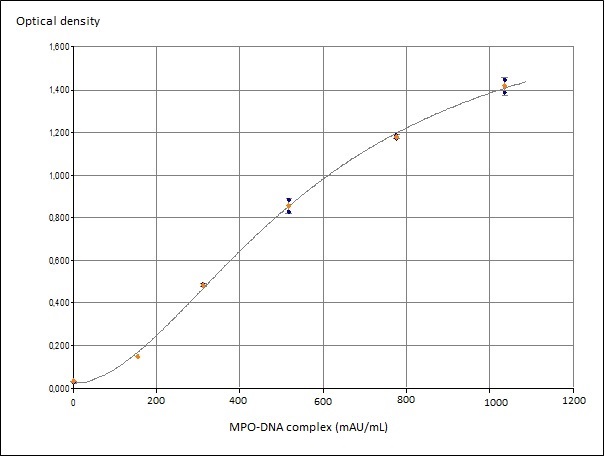
**

Curve of the reference line from an ELISA plate. Blood samples were measured in duplicates.

**S3 Fig. Correlation between MPO-DNA complex and citrullinated histone H3.**

Correlation between MPO-DNA complex (mAU/mL) and citrullinated histone H3 (ng/mL) in a random subset (n=35) of the general population.
