# Supplementary material for: Circulating Myeloperoxidase (MPO)-DNA complexes as marker for Neutrophil Extracellular Traps (NETs) levels and the association with cardiovascular risk factors in the general population
Source: PLoS One. 2021 Aug 11;16(8):e0253698. doi: 10.1371/journal.pone.0253698 (PMC8357174; doi:10.1371/journal.pone.0253698)
Supplement: Attachment — Submitted filename: Response to the reviewers.docx [file pone.0253698.s002.docx]

 Response to the reviewers

 Reviewer #1:

In this study, the authors aimed to assess whereas neutrophil extracellular trap (NET) formation is associated with demographic and/or cardiovascular risk factors in general population. NETs were quantified by measuring MPO-DNA complex using an ELISA. The results showed that MPO-DNA complex levels were lower with advancing age, a history of coronary heart disease, the use of lipid-lowering drugs and higher HDL-cholesterol. Although the pathophysiologic hypothesis is intriguing, this reviewer has some concerns:

1. The differences in MPO-DNA complex levels between subjects with and without cardiovascular risk factors were very small and the biological and clinical relevance seems forced.

*Thank you for this comment. We agree with the reviewer and throughout the revised manuscript we nuanced the relevance of the effect we found of CVD risk factors on NETs levels. In the results section we used the word ‘weak’ prior to the associations we found. We added some extra sentences in the discussion on page 18 lines 237-238:*

*‘Although these associations were significant, the effect of these determinants on NETs levels were only mild.’*

*Page 18 lines 242-243:*

*‘Although the effect was limited, we observed a small decrease in levels of MPO-DNA complex with advancing age, which was independent of the presence of comorbidities.’*

*Page 21-22 lines 333-336:*

*‘Although the associations we found in this study were significant, it is doubtful that these associations are of clinical relevance. This may suggest that NETs are more important in the acute phase of disease than in a steady state situation’.*

*In addition we deleted the paragraph on possible mechanisms supporting the associations we found between HDL, history of CHD and the use of lipid lowering drugs with NETs levels and changed it into the following paragraph on page 19 lines 260-271:*

*‘In this study, we found some weak associations between NETs and CVD risk factors, such as HDL, history of CHD and the use of lipid lowering drugs. The precise mechanisms behind these associations remain unknown. In case of HDL and lipid lowering drugs, a possible anti-inflammatory effect might play a role* [27, 28]*. In previous studies on patients with CVD, the role of NETs have already been well described. For instance, in patients with coronary atherosclerosis, high NETs levels were associated with major adverse cardiovascular events [4]. Also, in patients with an acute ST-elevation MI (STEMI), NETs are elevated in the acute phase.[27] The main difference between previous studies and our study, is that we measured NETs in a community dwelling population which cannot directly be compared to case-control studies in patients with active or acute disease. In addition, in case-control studies, specific patient groups are selected with certain disease characteristics and disease severities. In this study, such a selection was not present and individuals with minor disease as well as severe disease were all included.’*

*We also rephrased the conclusion of the manuscript on page 22, lines 340-344:*

*‘Older age, a history of coronary heart disease, the use of lipid-lowering drugs and higher HDL-cholesterol are weakly correlated with lower plasma levels of NETs. The findings of this study demonstrate that the effect of CVD risk factors on NETs levels in a general population is limited and may not be of clinical relevance. This emphasizes that NETs may play a more important role in an acute phase of disease than in a steady state situation.’*

2. In the Methods section, the definition of cardiovascular risk factors seems partial and incomplete, mainly for hypertension and hypercholesterolemia. The pharmacological therapy was only partially considered and the definition of “coronary heart disease” seems insufficient.

*We thank the reviewer for this suggestion. The definition of the CVD risk factors used in this study, were already defined at the beginning of the Rotterdam study in the nineties. To clarify the definition of CHD we added the following sentence on page 5 lines 105-107.*

*‘Coronary heart disease (CHD) was defined as fatal and non-fatal myocardial infarction and other coronary heart disease mortality. This includes myocardial infarction, myocardial revascularization, CHD mortality and overall CHD [15].’*

*We agree with the reviewer that we did not mention the definition of hypertension in our methods section, so we added this on page 5 lines 96-98:*

*‘We defined hypertension as a systolic blood pressure of 140 mmHg or higher, or a diastolic blood pressure of 90 mmHg or higher, or the use of blood pressure lowering medication.’*

*We did not define hypercholesterolemia, since we did not use the dichotomous variable hypercholesterolemia in our analyses. Instead we used the continues variables of total cholesterol and HDL. We therefore did not put a definition in our methods section.*

3. The model used for multivariable regression analysis only contemplate age and sex adjustment while other variables with a p value <0.1 at univariable analysis were not considered. Furthermore, there were no significant differences in MPO-DNA complex levels between male and female at univariate analysis.

*When searching for determinants in general, we found it not desirable to adjust for too many variables. This may lead to the inability to identify possible determinants. This is in contrast to disease risk studies, where it is common to adjust for possible confounders. Nevertheless, we added this important issue as a possible limitation to the discussion section on page 21 lines 329-333:*

*‘Another limitation of this study is the possibility of confounding factors. For this study, we were interested in identifying determinants of NETs levels. When adjusting for possible confounders, there is a risk of over-adjusting, resulting in the inability to identify possible determinants. This in in contrast to studies focusing on disease risk, where it is customary to adjust for possible confounders.’*

4. The discussion paragraph is too long. The argument is complex and sometimes farfetched and vulnerable.

*We understand this point of the reviewer. We therefore deleted the part on possible mechanisms supporting the associations we found between HDL, history of CHD and the use of lipid lowering drugs with NETs levels and changed it into the following paragraph on page 19 lines 260-271:*

*‘In this study, we found some weak associations between NETs and CVD risk factors, such as HDL, history of CHD and the use of lipid lowering drugs. The precise mechanisms behind these associations remain unknown. In case of HDL and lipid lowering drugs, a possible anti-inflammatory effect might play a role* [27, 28]*. In previous studies on patients with CVD, the role of NETs have already been well described. For instance, in patients with coronary atherosclerosis, high NETs levels were associated with major adverse cardiovascular events [4]. Also, in patients with an acute ST-elevation MI (STEMI), NETs are elevated in the acute phase.[27] The main difference between previous studies and our study, is that we measured NETs in a community dwelling population which cannot directly be compared to case-control studies in patients with active or acute disease. In addition, in case-control studies, specific patient groups are selected with certain disease characteristics and disease severities. In this study, such a selection was not present and individuals with minor disease as well as severe disease were all included.’*

*We also deleted the part on double stranded DNA, as it has no additional value to our study.*

Reviewer #2: In this manuscript, Donkel et al. evaluated the levels of NETs in a large cohort from the prospective population-based Rotterdam Study. The authors investigated the association between NET levels, measured by MPO-DNA ELISA, and different clinical, biochemical, cellular and inflammatory parameters. The study is certainly of interest given that this kind of study is missing and may give important answers on NET pathophysiology.

1. The main criticism that I have is on how the results are presented. The authors should be cautious with the conclusions. All the correlations are at most weak (max Rs is 0.11!) and the majority are very weak (<0.1). Thus, as the authors pointed it out in the limitation section, the biological, pathophysiological, and clinical relevance of these associations are very difficult to establish. The presence of confounding variables adds additional difficulty to interpret data.

*Thank you for this comment. We agree with the reviewer and throughout the manuscript we nuanced the relevance of the effect we found of CVD risk factors on NETs levels. In the results section we used the word ‘weak’ prior to the associations we found. We added some extra sentences in the discussion on page 18 lines 237-238:*

*‘Although these associations were significant, the effect of these determinants on NETs levels were only mild.’*

*Page 18 lines 242-243:*

*‘Although the effect was limited, we observed a small decrease in levels of MPO-DNA complex with advancing age, which was independent of the presence of comorbidities.’*

*Page 21-22 lines 333-336:*

*‘Although the associations we found in this study were significant, it is doubtful that these associations are of clinical relevance. This may suggest that NETs are more important in the acute phase of disease than in a steady state situation’.*

2. The authors should not be scared to present the manuscript focusing on that NETs are not or very weakly associated with the different parameters since this study is very informative regarding a healthy population. In this sense, the abstract gives a message that is not conveyed once the manuscript is read. In particular, the conclusions of the abstract and of the manuscript should be rephrased and soften. The discussion should be reduced since several very weak associations do not need so much discussion.

*We agree with the reviewer. And as can be seen from the our response to the previous question, we softened our statements about the found associations and we rephrased our abstract and manuscript conclusions on page 2, lines 37-41 and page 22, lines 340-344:*

*‘Older age, a history of coronary heart disease, the use of lipid-lowering drugs and higher HDL-cholesterol are weakly correlated with lower plasma levels of NETs. The findings of this study demonstrate that the effect of CVD risk factors on NETs levels in a general population is limited and may not be of clinical relevance. This emphasizes that NETs may play a more important role in an acute phase of disease than in a steady state situation.’*

*To shorten the discussion, we deleted the part on possible mechanisms supporting the associations we found between HDL, history of CHD and the use of lipid lowering drugs with NETs levels and changed it into the following paragraph on page 19 lines 260-271:*

*‘In this study, we found some weak associations between NETs and CVD risk factors, such as HDL, history of CHD and the use of lipid lowering drugs. The precise mechanisms behind these associations remain unknown. In case of HDL and lipid lowering drugs, a possible anti-inflammatory effect might play a role* [27, 28]*. In previous studies on patients with CVD, the role of NETs have already been well described. For instance, in patients with coronary atherosclerosis, high NETs levels were associated with major adverse cardiovascular events [4]. Also, in patients with an acute ST-elevation MI (STEMI), NETs are elevated in the acute phase.[27] The main difference between previous studies and our study, is that we measured NETs in a community dwelling population which cannot directly be compared to case-control studies in patients with active or acute disease. In addition, in case-control studies, specific patient groups are selected with certain disease characteristics and disease severities. In this study, such a selection was not present and individuals with minor disease as well as severe disease were all included.’*

*We also deleted the part on double stranded DNA, as it has no additional value to our study.*

3. This work has to show its strengths, that are the casuistic and the large amount of parameters to perform the statistics. The authors may give conclusions concerning the lack of associations more than the presence of associations between NETs and different parameters.

*We added a passage on the strengths of this study in the discussion on page 21, lines 318-322:*

*‘The main strength of this study, is that this is the first study that measured MPO-DNA complex levels in a very large community dwelling population and investigated the association with several known CVD risk factors. Although we were unable to demonstrate a clinical relevant association between NETs and any of the CVD risk factors, the findings of this study are still of importance to identify the role of NETs on population level.’*

*We also provided conclusions on the lack of association as can be seen previously.*

Beside this main concern, other minor points should be attended:

• Pleas e check for typo errors (e.g. line 117: phorbol 12-myristaat 13-acetaat)

*Thank you for pointing this out. We changed is to phorbol 12-myristate 13-acetate.*

• The concentration of PMA used to activate NETosis has to be indicated in M&M

*We added the concentration PMA in line 124 on page 6: ‘250 ng/mL phorbol 12-myristate 13-acetate (PMA) (stock 100 µg/mL in DMSO)’*

• Line 191, please add the reference to Figure 1

*When uploading our manuscript. We had to embed the title and figure legend in the manuscript text. The figure had to be uploaded separately without caption and legend. For clarity, we added the figure with title and legend below.*

**Fig 1. Distribution of MPO-DNA complex levels among age categories**

*p<0.01. Data are presented as median and 25^th^-75^th^ percentiles. Category 55-65 years, (n=2286) MPO-DNA complex 56 mAU/mL (45-93), category 65-75 years (n=2467) MPO-DNA complex 53 mAU/mL (41-83), category >75 years (n=1674) MPO-DNA complex 52 AU/mL (41-81). Differences between age categories were analyzed using the Kruskall –Wallis test with post-hoc analysis.

• Line 313. Authors should moderate the sentence given that other studies have shown a correlation between MPO/DNA and cfDNA (e.g. 31119471 (septic patients), 32329756 (Covid-19))

*We deleted the part on cfDNA to shorten the discussion. Furthermore, we found that the part on cfDNA was not of additional value in our discussion.*

• Line 326. This result is important and should be shown.

*We added a graph of the correlation between MPO-DNA complex and CitH3 in the supplemental material.*

**Supplemental figure 3. Correlation between MPO-DNA complex and citrullinated histone H3.**

Citrullinated H3 ng/ml

| MPO-DNA complex mAU/ml | Citrullinated H3 (ng/ml) |
| --- | --- |
| 813 | 0.78 |
| 550 | 1.49 |
| 178 | 1.68 |
| 54 | 1.11 |
| 60 | 0.51 |
| 939 | 1.87 |
| 28 | 1.1 |
| 50 | 1.61 |
| 463 | 0.9 |
| 468 | 2.11 |
| 40 | 1.13 |
| 162 | 7.32 |
| 40 | 2.44 |
| 45 | 1.25 |
| 1025 | 0.98 |
| 560 | 0.39 |
| 94 | 3.81 |
| 67 | 2.77 |
| 866 | 0 |
| 53 | 0.1 |
| 376 | 1.25 |
| 715 | 1.46 |
| 114 | 1.75 |
| 38 | 0.68 |
| 30 | 3.28 |
| 292 | 1.49 |
| 50 | 0.9 |
| 433 | 1.08 |
| 6 | 3.36 |
| 16 | 9.13 |
| 10 | 3.25 |
| 19 | 1.58 |
| 89 | 1.21 |
| 50 | 1.87 |
| 77 | 1.46 |

Correlation between MPO-DNA complex (mAU/mL) and citrullinated histone H3 (ng/mL) in a random subset (n=35) of the general population.

• Significant correlations have to be shown in a graph.

*We agree that it would be interesting to show these data in graphs. However, since the high number of measurements and the very weak correlations, the graphs were not informative. As can be seen in the example below of graph including MPO-DNA complex and HDL levels.*

• How do the authors explain the lower levels of NETs in older subjects? Can this be associated with drug intake that can be higher in older vs younger individuals?

*This is indeed very interesting. We hypothesize that this might be explained by immunosenescence of neutrophils in the elderly. In previous studies, it has been demonstrated that the function of neutrophils decreases with increasing age. We think that this may also be the case in the formation of NETs.*

*We added our hypothesis in the discussion on page 18-19 lines 255-259:*

*‘It is known that the elderly have an overall increased susceptibility to infection and also have a suboptimal immune response after vaccination [23-25]. Immunosenescence is part of the aging process and also effects neutrophil function [26]. Since neutrophil function decreases with age, the formation of NETs could possibly also be decreased.’*
